# COVID-19 Vaccination and Remdesivir are Associated With Protection From New or Increased Levels of Donor-Specific Antibodies Among Kidney Transplant Recipients Hospitalized With COVID-19

**DOI:** 10.3389/ti.2022.10626

**Published:** 2022-07-19

**Authors:** John T. Killian, Julie A. Houp, Greer A. Burkholder, Salomon A. Roman Soto, A. Cozette Killian, Song C. Ong, Nathaniel B. Erdmann, Paul A. Goepfert, Vera Hauptfeld-Dolejsek, Sixto M. Leal, Esther Zumaquero, Anoma Nellore, Gaurav Agarwal, Clifton E. Kew, Babak J. Orandi, Jayme E. Locke, Paige M. Porrett, Emily B. Levitan, Vineeta Kumar, Frances E. Lund

**Affiliations:** ^1^ Department of Surgery, Heersink School of Medicine, University of Alabama at Birmingham, Birmingham, AL, United States; ^2^ Department of Medicine, Heersink School of Medicine, University of Alabama at Birmingham, Birmingham, AL, United States; ^3^ Department of Pathology, Heersink School of Medicine, University of Alabama at Birmingham, Birmingham, AL, United States; ^4^ Department of Microbiology, Heersink School of Medicine, University of Alabama at Birmingham, Birmingham, AL, United States; ^5^ School of Public Health, University of Alabama at Birmingham, Birmingham, AL, United States

**Keywords:** COVID-19, kidney transplantation, alloimmunity, donor-specific antibodies, vaccination

## Abstract

Alloimmune responses in kidney transplant (KT) patients previously hospitalized with COVID-19 are understudied. We analyzed a cohort of 112 kidney transplant recipients who were hospitalized following a positive SARS-CoV-2 test result during the first 20 months of the COVID-19 pandemic. We found a cumulative incidence of 17% for the development of new donor-specific antibodies (DSA) or increased levels of pre-existing DSA in hospitalized SARS-CoV-2-infected KT patients. This risk extended 8 months post-infection. These changes in DSA status were associated with late allograft dysfunction. Risk factors for new or increased DSA responses in this KT patient cohort included the presence of circulating DSA pre-COVID-19 diagnosis and time post-transplantation. COVID-19 vaccination prior to infection and remdesivir administration during infection were each associated with decreased likelihood of developing a new or increased DSA response. These data show that new or enhanced DSA responses frequently occur among KT patients requiring admission with COVID-19 and suggest that surveillance, vaccination, and antiviral therapies may be important tools to prevent alloimmunity in these individuals.

## GRAPHICAL ABSTRACT

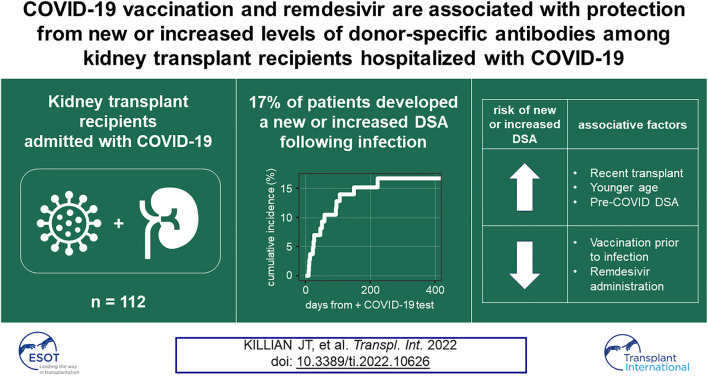

## Introduction

SARS-CoV-2 infection, which elicits acute COVID-19 clinical disease, has been devastating for kidney transplant (KT) recipients (1, 2). Compared to non-solid organ transplant (nonSOT) patients, KT recipients (KTRs) are more likely to be hospitalized and experience greater COVID-19-related morbidity and mortality (3). Given the estimated 17,000 KTRs that have been admitted with COVID-19 in the United States (4), it is critically important to characterize acute and chronic effects of COVID-19.

A recent report showed that 8% of KTRs hospitalized with COVID-19 developed a new donor-specific antibody (DSA) response or exhibited increased levels of a known pre-existing DSA within a median follow-up of 45 days post-infection (5). Given inpatient reductions in immunosuppressive therapy (5), the protracted immune response to COVID-19 (6), the impaired ability to achieve SARS-CoV-2 viral clearance among immunosuppressed individuals (7), and evidence of late allograft dysfunction following COVID-19 (8, 9), we hypothesized that KTRs may suffer alloimmune consequences from COVID-19 that extend well beyond the acute phase of infection.

To test this hypothesis, we evaluated alloimmune responses in KTRs hospitalized following SARS-CoV-2 infection. In addition, we identified risk factors associated with alloimmunity and allograft dysfunction. We found that the risk of alloimmunity in KTRs extended at least 8 months past admission for COVID-19 and observed that new or increased DSA responses were associated with decreased late allograft function. Vaccination and antiviral therapies were each associated with a reduced risk of a new or increased DSA response, suggesting that alloimmune responses in KTRs may be regulated directly or indirectly by SARS-CoV-2 infection.

## Materials and Methods

### Cohort and Design

We performed a single-center prospective observational cohort study of KTRs hospitalized with COVID-19. Patients admitted to the University of Alabama at Birmingham (UAB) Hospital between 1 March 2020 and 1 November 2021 with a positive SARS-CoV-2 reverse transcriptase polymerase chain reaction or antigen test that occurred within 14 days prior to or 7 days after the day of admission were approached for enrollment. For patients with multiple COVID-19-associated admissions, only the first hospitalization was considered. Participants or, in cases of incapacitation, legally authorized representatives provided consent. The UAB Institutional Review Board approved the study protocol (IRB-300005127).

### Data Collection

We obtained data on demographics, comorbidities, medications, transplant surgical history, inpatient treatment, and outcomes. Data were extracted from the electronic medical record and transformed. We obtained all anti-HLA antibody (HLA-Ab) and renal function studies extending from 14 months prior to COVID-19 diagnosis through 1 February 2022. HLA-Ab testing was supplemented with available research biospecimens as described below.

### Outcome Assessments and Definitions

The primary outcome analyzed was the development of new DSA or increased levels of pre-existing DSA at least 10 days after COVID-19 diagnosis. DSA not previously present that crossed the 1500 mean fluorescence intensity (MFI) threshold was classified as new DSA. Although these new DSAs were also by definition *de novo* (10), we use the term *new* to indicate the appearance of the anti-HLA specificity post-COVID-19, as opposed to other patients who had *stable de novo* DSAs present both pre-COVID-19 and post-COVID-19. Increased DSA was defined as DSA that rose >1000 MFI and represented a >25% increase over baseline MFI. For patients with a history of multiple transplants, only DSA targeting HLA expressed by the functioning kidney allograft was considered. To adjudicate DSA responses, all patients required an HLA-Ab measurement within 14 months prior to the measurement with increased MFI. For most samples, HLA-Ab testing was performed for-cause and not by a prescribed protocol. In addition to HLA-Ab testing ordered by a clinician, we analyzed research serum samples collected in accordance with study protocols. We did this to establish baseline HLA-Ab measurements for patients lacking pre-COVID-19 samples and to evaluate DSA responses ≥10 days after COVID-19 diagnosis in patients without clinician-ordered HLA-Ab testing. For both HLA class I and class II, the antibody specificity with the maximum MFI across all timepoints was classified as immunodominant.

The secondary outcome analyzed was a 30% decline from the baseline estimated glomerular filtration rate (eGFR) ≥90 days from COVID-19 diagnosis (11). Two consecutive eGFR measurements below 70% of the baseline eGFR, with no subsequent eGFR recovery, defined the outcome. The Chronic Kidney Disease Epidemiology Collaboration (CKD-EPI) equation was used to calculate eGFR (12). Baseline eGFR was defined as the median eGFR among measurements from 365 days (or if transplanted in the year prior to COVID-19 diagnosis, from the date of post-transplant eGFR stabilization) to 7 days before COVID-19 diagnosis.

Additional outcomes analyzed included the following: Acute kidney injury (AKI) was classified using Kidney Disease Improving Global Outcomes (KDIGO) stages (13). Serum creatinine (sCr) values, used for eGFR calculations, were winsorized at the 95th percentile (14), which was 5.9 mg/dl for this study. If a patient experienced allograft failure, subsequent sCr measures were censored, and sCr at time of allograft failure was set equal to 5.9 mg/dl. Patients were defined as being vaccinated if they had received at least one dose of a COVID-19 vaccine prior to admission. World Health Organization (WHO)-defined COVID-19 disease severity was scored from 3 (admission without supplemental oxygen requirement) to 8 (death) (15).

Inpatient reduction of immunosuppression was calculated as the percentage reduction of each outpatient immunosuppressive dose. This reduction was calculated by dividing the total inpatient administered dose of each medication by the total expected dose based upon the outpatient maintenance regimen documented by the admitting physician, excluding inpatient medications administered on the day of admission or discharge. Corticosteroid doses were transformed into prednisone equivalent doses based upon relative potencies (16). Remdesivir was administered per institutional protocols for patients requiring supplemental oxygen and with eGFR >30 mL/min per 1.73 m^2^ and serum alanine transferase <260 units/L.

### Statistical Analyses

Categorical variables were presented as count and percentage. Continuous variables were presented with median and interquartile range (IQR). Pearson’s Chi-squared test was used to compare percentages between categorical variables when all expected cell counts were ≥5, and Fisher’s exact test was used when any expected cell count was <5. Group mean ranks were compared with Mann-Whitney tests. Differences in matched samples were compared using the Wilcoxon matched-pairs signed rank test. Multiple comparisons of group mean ranks were performed using the Friedman test adjusted with Dunn’s multiple comparisons. An alpha of 0.05 was used as the cutoff for statistical significance. All tests were two-tailed.

The two main outcomes analyzed, development of new or increased levels of DSA and the loss of 30% of baseline eGFR, were estimated using the Kaplan-Meier (KM) method. eGFR loss was estimated using a landmark analysis, starting at a landmark of 90 days after COVID-19 diagnosis (17). Time-to-event analyses were censored by death and the end of the study follow-up period. The logrank test was used to evaluate for statistical differences in the probability of the outcome of interest at any time point. All statistical analyses were performed using R version 4.0.2 and GraphPad Prism version 9.3.1.

## Results

### Cohort Characteristics

**FIGURE 1:**
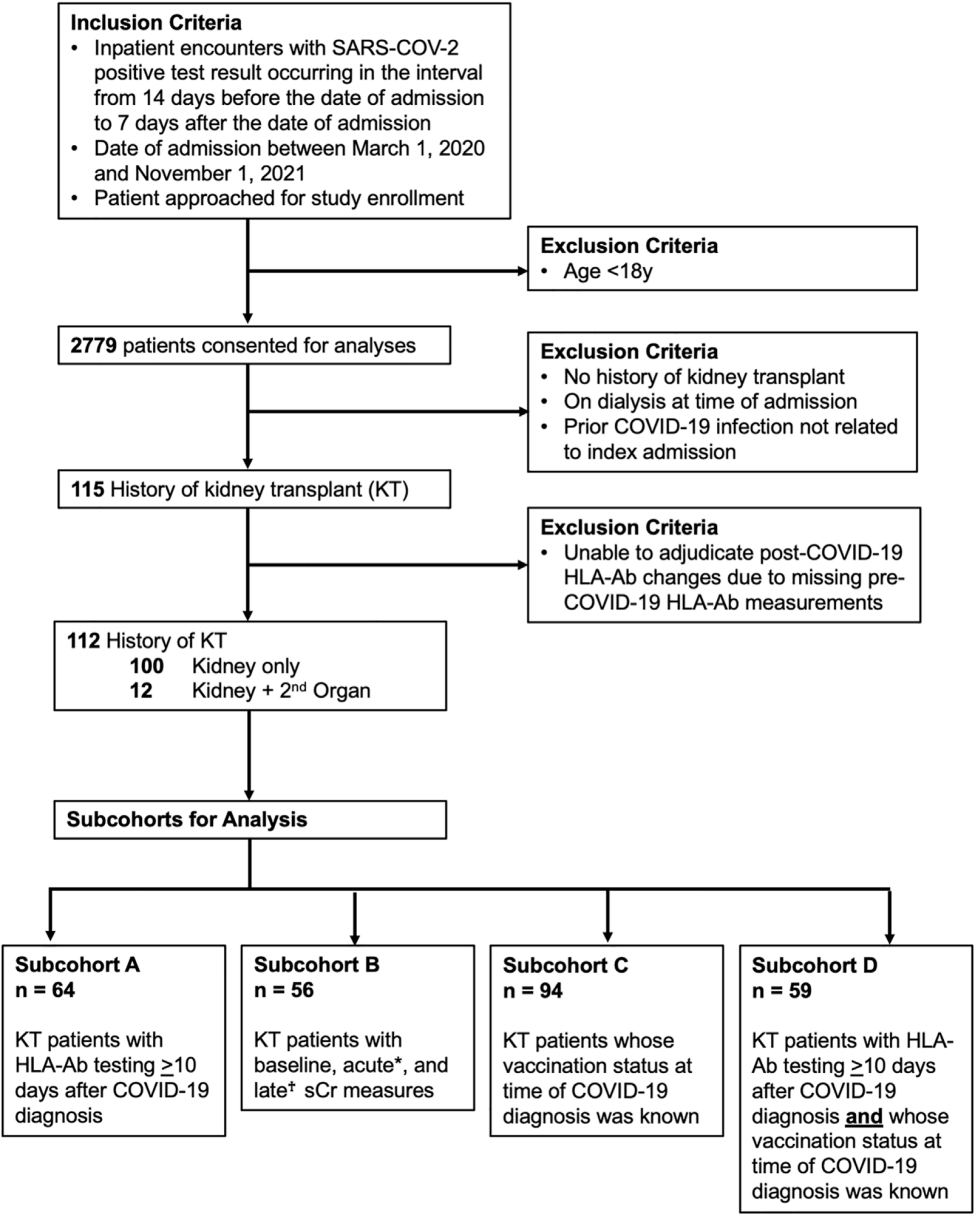
Cohort inclusion criteria and data availability. Flowchart for included participants and four subcohorts used for analyses. sCr indicates serum creatinine. KT indicates kidney transplant. HLA-Ab indicates anti-HLA antibody. *Acute sCr refers to measures occurring both 1) during the index admission and 2) within 30 days of the COVID-19 diagnosis. ^†^Late sCr refers to measures occurring at least 90 days after the COVID-19 diagnosis.

We prospectively enrolled KTRs hospitalized with a COVID-19 diagnosis at UAB between March 1, 2020 and November 1, 2021 (Figure 1). Of 115 consented KTRs with functioning allografts, 3 patients for whom DSA adjudication was impossible were excluded, yielding 112 KTRs for analysis (Table 1). 64 (57%) patients had at least one HLA-Ab test ≥10 days after their COVID-19 diagnosis (subcohort A). Baseline HLA-Ab testing was available at a median of 66 days prior to COVID-19 diagnosis (IQR 15–110 days). For a single patient, the baseline HLA-Ab measure was conducted over 365 days prior to COVID-19 diagnosis (at 414 days before COVID-19), and this patient had been followed regularly with clinic visits and stable allograft function for the year prior to COVID-19. These 64 patients had a total of 197 HLA-Ab tests (on average, 3.1 tests per patient). 175 of these tests were ordered by the attending transplant nephrologist, and 22 were research serum specimens that were subsequently tested in the HLA laboratory (Supplementary Figure S1). 56 (50%) patients (subcohort B) had baseline, acute, and late (≥90 days from COVID-19 diagnosis) sCr measurements. 94 (84%) patients had a known COVID-19 vaccination status at the time of admission (subcohort C), and 59 (53%) patients had both HLA-Ab testing ≥10 days after COVID-19 diagnosis and known COVID-19 vaccination status (subcohort D).

**TABLE 1:** Overview of cohort demographics, transplant history, and disease severity.

	N = 112
**Demographics**	
**Age**	56 (49, 64)
**Gender**	
Female	45 (40%)
Male	67 (60%)
**Race**	
Black	57 (51%)
Other	3 (2.7%)
White	52 (46%)
**Ethnicity**	
Hispanic	3 (2.7%)
Non-Hispanic	109 (97%)
**Transplant-related history**	
**Type of transplant**	
Kidney	100 (89%)
Kidney, Heart	1 (0.9%)
Kidney, Liver	4 (3.6%)
Kidney, Lung	1 (0.9%)
Kidney, Pancreas	6 (5.4%)
**Time from kidney transplant <1 year**	31 (28%)
**Presence of DSA pre-COVID-19**	12 (19%)
Unknown	48
**Disease Severity**	
**Highest WHO COVID-19 disease severity scale**	
3 (no supplemental oxygen)	17 (15%)
4 (supplemental oxygen via nasal cannula)	38 (34%)
5 (supplemental oxygen via high-flow nasal cannula, BiPap, or CPAP)	19 (17%)
6 (endotracheal intubation and mechanical ventilation)	5 (4.5%)
7 (endotracheal intubation and mechanical ventilation + vasopressor support or ECMO)	3 (2.7%)
8 (death)	30 (27%)

Cell values presented as median (IQR) for continuous variables and n (%) for categorical variables. DSA, donor-specific antibody; WHO, World Health Organization; ECMO, extracorporeal membrane oxygenation.

### New or Increased Donor-Specific Antibody Responses Were Frequently Observed in Kidney Transplant Patients Following Admission for COVID-19

**FIGURE 2:**
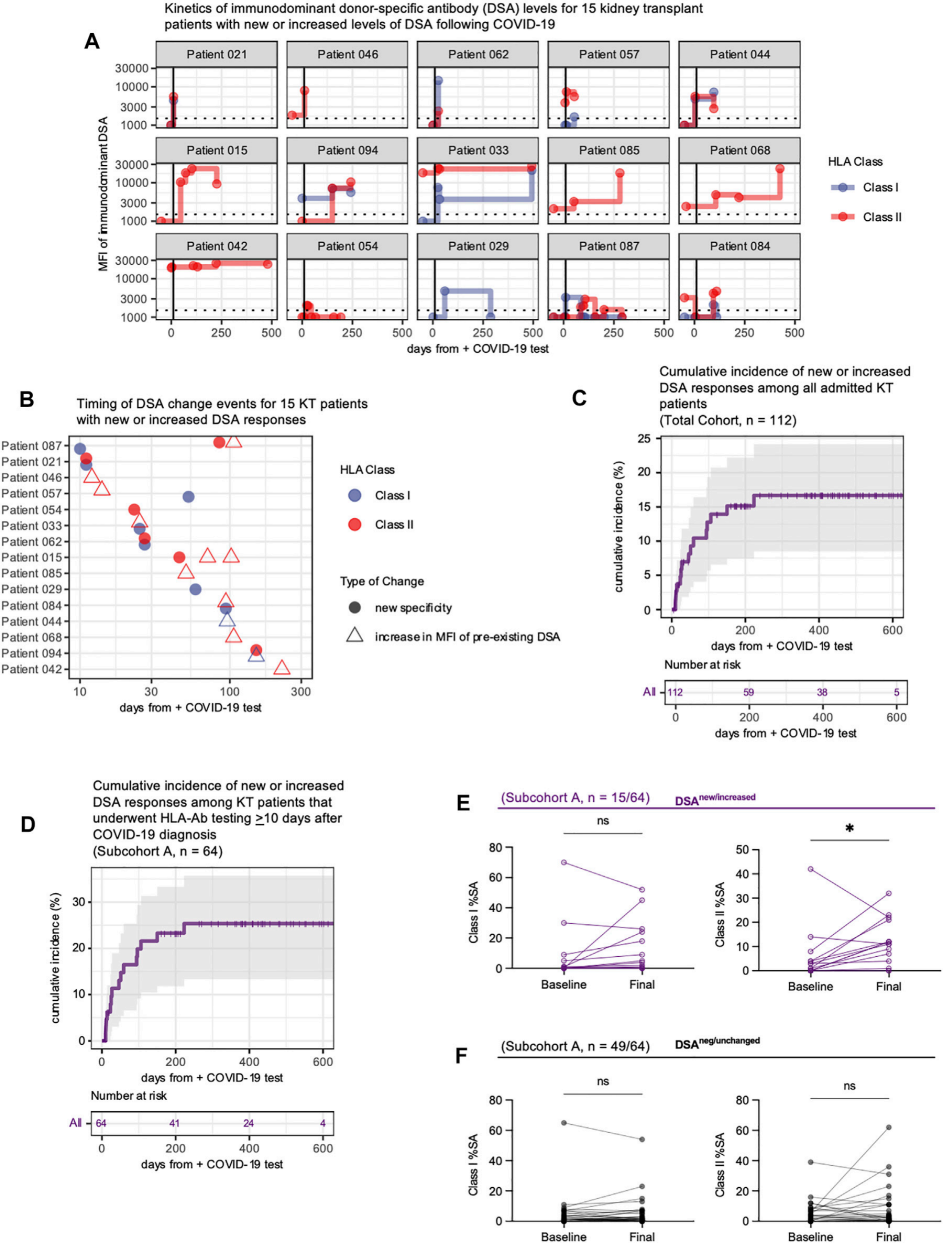
COVID-19 infection is associated with development of new or increased DSA responses for up to 8 months. **(A)** Longitudinal assessment of the immunodominant class I and/or class II DSA levels for 15 patients who developed new or increased DSA responses following diagnosis of COVID-19. Each facet plot shows a single patient. DSA responses are shown as the mean fluorescence intensity (MFI) of binding by serum antibodies to the HLA bead array. The horizontal dashed line indicates the laboratory threshold for a positive DSA (MFI = 1500). Results below this MFI threshold are plotted at MFI = 1000. The vertical black line indicates 10 days after COVID-19 diagnosis. Any baseline measurement that occurred greater than 50 days before the COVID-19 diagnosis is plotted at x = −50 days. **(B)** The timing of the DSA changes shown in **A**, plotted based on DSA reactivity to individual HLA class I (blue symbols) or class II (red symbols) antigens, and whether the DSA was reactive to a new specificity (filled circle) or represented an increase in a preexisting DSA response (open triangle). **(C,D)** Cumulative incidence of new or increased DSA for the entire hospitalized COVID-19 KT patient cohort [*n* = 112, panel **(C)**] or those individuals with HLA-Ab testing ≥10 days after COVID-19 diagnosis [subcohort A, *n* = 64, panel **(D)**]. Incidence defined as the development of a new DSA specificity or an increase in a DSA specificity (MFI change >1000 and an overall increase of >25%). Patients are censored at time of death or at the end of the study follow-up period. **(E,F)** Analysis of breadth of anti-HLA reactivity in patient serum collected pre-COVID-19 (Baseline) and at the final measurement post-COVID-19 (Final). Samples were analyzed against a full panel of microbeads coated with single HLA class I or class II antigens. The percentage of positive (defined as an MFI ≥4 standard deviations above the background MFI) single antigen beads (%SA) for each sample was determined. Data shown are for patients in subcohort A with %SA measurements at both timepoints and include patients (*n* = 15) with new or increased DSA levels **(E)** and patients (*n* = 49) whose DSA responses were negative or unchanged **(F)**. Data were analyzed using the Kaplan-Meier method **(C,D)** or the Wilcoxon matched-pairs signed rank test **(E,F)**. ns, *p* > 0.05; *p ≤ 0.05, **p < 0.01; ***p < 0.001, ****p < 0.0001.

Fifteen patients developed new or increased DSA responses at ≥10 days following COVID-19 diagnosis (Figure 2A). Ten of fifteen (67%) patients developed a new DSA specificity not present pre-COVID-19 (defined as increasing above a 1500 MFI threshold), while five had increases (defined as an increase of both >1000 MFI and >25% from baseline MFI) in pre-existing DSA levels (Figure 2A, Supplementary Table S1). Four patients showed subsequent resolution of at least one DSA specificity within 1 year of COVID-19 diagnosis, but for all other patients, DSA responses persisted (Figure 2A). Development of new or increased DSA occurred within 6 months of COVID-19 diagnosis for 14/15 (93%) patients (Figure 2B). Within the entire cohort of 112 KTRs, which included those with and without post-COVID HLA-Ab testing, the cumulative incidence of new or increased DSA responses was 17% at 230 days post-COVID-19 (Figure 2C). When we restricted our analysis to the subcohort of patients with post-COVID-19 HLA-Ab testing (subcohort A, *n* = 64), we calculated the cumulative incidence to be 25% at 230 days post-COVID-19 (Figure 2D). Thus, we concluded that a minimum of 17% of admitted KTRs in our study developed a new or increased DSA response within 8 months following admission with COVID-19.

We then asked whether this DSA response developed in the context of a broad anti-HLA response. To assess the breadth of anti-HLA reactivity we measured reactivity of serum samples against an array of HLA class I and class II single antigen (SA) beads prior to COVID-19 diagnosis and at the final observation post-COVID-19. At each timepoint, we determined the percentage of HLA class I and class II antigens (%SA) that were bound by donor serum (defined as MFI ≥4 standard deviations above background MFI). Patients with new or increased DSA responses showed an increase in the breadth of anti-class II responses (Figure 2E). However no other changes in the breadth of anti-HLA reactivity were statistically significant, and patients with negative or unchanged DSA responses had no significant increase in the breadth of anti-HLA reactivity (Figure 2F). Thus, the alloimmune response that develops following COVID-19 appears to be donor-specific and not merely the result of COVID-19 eliciting a broadly reactive anti-HLA response.

### Kidney Transplant Patients With New or Increased Donor-Specific Antibody Responses Had Distinguishing Clinical Features

Our data showed new or increased DSA responses in a substantial fraction of KTRs hospitalized with COVID-19. To assess whether this alloimmune response was associated with specific clinical features, we assessed demographics, comorbidities and transplant history using an unadjusted bivariate analysis. In Table 2, we show that the 15 patients with new or increased DSA levels were younger, more likely to have been recently transplanted (53% vs. 24%, *p* = 0.028), and more likely to have pre-COVID-19 DSA (47% vs. 10%, *p* = 0.004). By contrast, other baseline demographics and comorbidities were similar between groups. Thus, younger age, pre-COVID-19 DSA and recency of transplant were associated with the development of new or increased DSA levels.

**TABLE 2:** Comparison of demographics, comorbidities, and transplant-related history based upon the development of new or increased DSA responses following COVID-19.

	DSA negative/unchanged/unknown, N = 97	DSA new/increased, N = 15	*p*-value
**Demographics**			
** Age**	57 (50, 64)	48 (40, 55)	0.010
** Gender**			0.3
Female	41 (42%)	4 (27%)	
Male	56 (58%)	11 (73%)	
**Race**			0.067
Black	45 (46%)	12 (80%)	
Other	3 (3.1%)	0 (0%)	
White	49 (51%)	3 (20%)	
**Ethnicity**			>0.9
Hispanic	3 (3.1%)	0 (0%)	
Non-Hispanic	94 (97%)	15 (100%)	
**Comorbidities**			
**Diabetes mellitus**	67 (69%)	12 (80%)	0.5
**COPD**	13 (13%)	3 (20%)	0.4
**Hypertension**	94 (97%)	14 (93%)	0.4
**Coronary artery disease**	38 (39%)	5 (33%)	0.7
**Congestive heart failure**	33 (34%)	6 (40%)	0.7
**Never smoker**	27 (28%)	1 (6.7%)	0.11
**Obesity**			0.5
Not obese	54 (56%)	7 (47%)	
Obese	43 (44%)	8 (53%)	
**Transplant-related history**			
**Type of transplant**			0.3
Kidney	87 (90%)	13 (87%)	
Kidney, Heart	1 (1.0%)	0 (0%)	
Kidney, Liver	4 (4.1%)	0 (0%)	
Kidney, Lung	0 (0%)	1 (6.7%)	
Kidney, Pancreas	5 (5.2%)	1 (6.7%)	
**Time from kidney transplant (days)**	1,496 (475, 2,958)	145 (100, 2,324)	0.068
**Time from kidney transplant <1 year**	23 (24%)	8 (53%)	0.028
**Presence of DSA pre-COVID-19**	5 (10%)	7 (47%)	0.004
Unknown	48	0	

Cell values presented as median (IQR) for continuous variables and n (%) for categorical variables. Pearson’s Chi-squared test was used to compare percentages between categorical variables when all expected cell counts were ≥5, and Fisher’s exact test was used when any expected cell count was <5. Group mean ranks were compared with Mann-Whitney tests. DSA, donor-specific antibody. COPD, chronic obstructive pulmonary disease.

Next, we asked whether disease severity was associated with new or increased DSA responses. In Table 3, we show that length of stay, level of care, mortality, and the WHO COVID-19 disease severity score were all similar between the 15 KTRs who experienced DSA changes post-COVID-19 diagnosis and those without DSA changes. Overall, baseline and acute allograft function were similar between groups. Although rates and severity of AKI were similar between groups, patients with new or increased DSA levels showed significantly greater decreases in eGFR at late timepoints (Table 3). Sensitivity analyses of KTRs with baseline, acute and late serum creatine (sCr) measurements (subcohort B, Supplementary Table S2) and subcohort B patients who also had post-COVID-19 HLA-Ab testing (Supplementary Table S3) confirmed those findings. Thus, while acute clinical severity was similar between groups, patients with new or increased DSA levels exhibited greater loss of eGFR at ≥90 days post-COVID-19.

**TABLE 3:** Comparison of disease severity and renal function based upon the development of new or increased DSA responses following COVID-19.

	DSA negative/unchanged/unknown, N = 97	DSA new/increased, N = 15	*p*-value
**Measures of disease severity**			
**Length of stay (days)**	10 (4, 19)	11 (4, 17)	>0.9
**ICU requirement**	40 (41%)	7 (47%)	0.7
**Highest WHO COVID-19 disease severity scale**			0.13
3 (no supplemental oxygen)	14 (14%)	3 (20%)	
4 (supplemental oxygen via nasal cannula)	34 (35%)	4 (27%)	
5 (supplemental oxygen via high-flow nasal cannula, BiPap, or CPAP)	17 (18%)	2 (13%)	
6 (endotracheal intubation and mechanical ventilation)	2 (2.1%)	3 (20%)	
7 (endotracheal intubation and mechanical ventilation + vasopressor support or ECMO)	3 (3.1%)	0 (0%)	
8 (death)	27 (28%)	3 (20%)	
**Discharge disposition**			0.7
Expired	27 (28%)	3 (20%)	
Home	59 (61%)	11 (73%)	
Long-term care facility	11 (11%)	1 (6.7%)	
**Renal function**			
**AKI Grade (KDIGO)**			0.3
0	20 (24%)	3 (23%)	
1	34 (41%)	4 (31%)	
2	5 (6.0%)	3 (23%)	
3	24 (29%)	3 (23%)	
Unknown	14	2	
**CRRT/HD while inpatient**	22 (23%)	3 (20%)	>0.9
**Baseline eGFR (mL/min/1.73 m^2^)**	43 (33, 61)	56 (41, 63)	0.3
Unknown	21	4	
**Absolute change in eGFR on 90+ day follow-up**	1 (−8, 7)	−14 (−17, −5)	0.013
Unknown	51	5	
**Percentage change in eGFR on 90+ day follow-up**	4 (−19, 17)	−29 (−56, −9)	0.005
Unknown	51	5	
**At least 30% loss in eGFR on 90+ day follow-up**			0.027
<30%	42 (91%)	6 (60%)	
≥30%	4 (8.7%)	4 (40%)	
Unknown	51	5	

Cell values presented as median (IQR) for continuous variables and n (%) for categorical variables. Pearson’s Chi-squared test was used to compare percentages between categorical variables when all expected cell counts were ≥5, and Fisher’s exact test was used when any expected cell count was <5. Group mean ranks were compared with Mann-Whitney tests. DSA, donor-specific antibody. WHO, World Health Organization^*.*^ ECMO, extracorporeal membrane oxygenation. AKI, acute kidney injury. KDIGO, Kidney Disease Improving Global Outcomes. CRRT, continuous renal replacement therapy. HD, hemodialysis. eGFR, estimated glomerular filtration rate.

### New or Increased Donor-Specific Antibody Was Associated With Late Loss of Allograft Function

**FIGURE 3:**
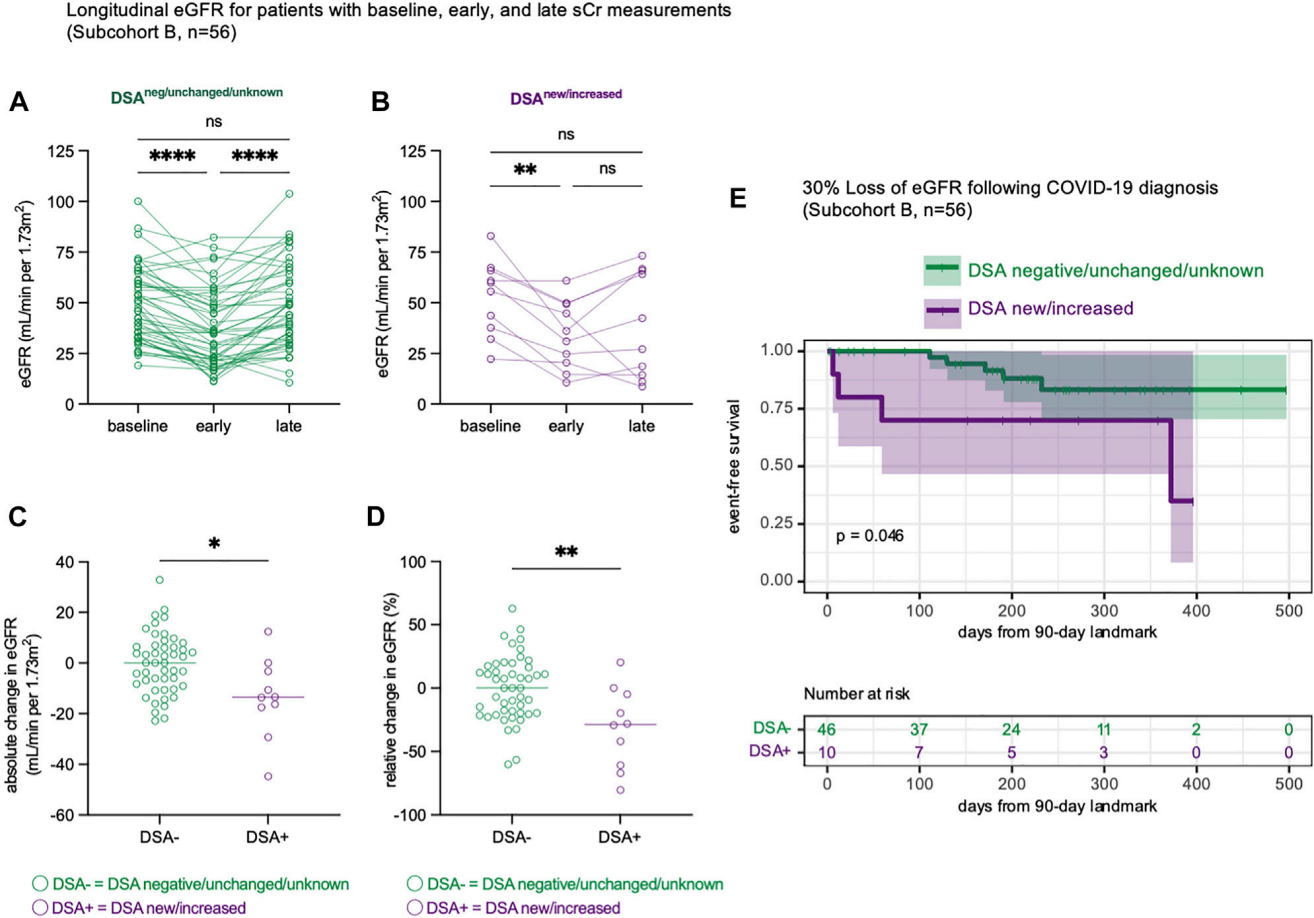
Late renal function is impaired in KT patients with new or increased DSA responses. **(A,B)** Estimated glomerular filtration rate (eGFR) trajectories for *n* = 56 patients (subcohort B) with baseline, early and late eGFR measurements. The baseline measurement represents the median eGFR in the year prior to COVID-19 infection. The early measurement represents the nadir eGFR during the hospital admission for COVID-19. The late measurement represents the final eGFR measurement that occurred at least 90 days following COVID-19 diagnosis. Data shown as eGFR measurements in patients whose DSA responses were negative, unchanged, or unknown [**(A)**, *n* = 46] and in patients with new or increased DSA responses [**(B)**, *n* = 10]. **(C)** The absolute change in eGFR from each patient’s baseline eGFR to their final eGFR, at least 90 days after COVID-19 diagnosis. **(D)** The relative change in eGFR from each patient’s baseline eGFR to their final eGFR, calculated as a percentage change of the baseline eGFR. **(E)** A time-to-event analysis modeling the late loss of at least 30% of baseline eGFR, starting at 90 days after COVID-19 infection. This is performed as a landmark analysis, where x = 0 is landmarked to 90 days after COVID-19 diagnosis. Statistical analyses were performed using Friedman’s test with the application of Dunn’s multiple comparisons test **(A,B)**, Mann-Whitney Test **(C,D)** or the logrank test **(E)**. ns, *p* > 0.05; *p ≤ 0.05, **p < 0.01; ***p < 0.001, ****p < 0.0001.

To better understand the progression of renal dysfunction post-COVID-19, we analyzed the change in eGFR over time, comparing patients with new or increased DSA levels to patients whose DSA levels were negative/unchanged/unknown (subcohort B, *n* = 56). Among the subset of patients with baseline, early inpatient, and late eGFR measurements, there was a significant decline in eGFR during admission (Figures 3A,B), with subsequent late recovery for many patients (Figures 3A,B). We then measured the absolute (Figure 3C) and relative (Figure 3D) changes in eGFR and found that patients with new or increased DSA levels had significantly greater late loss of eGFR. To estimate the incidence of a clinically meaningful (11, 18) late 30% loss of baseline eGFR, we performed a time-to-event analysis. This confirmed that patients with new or increased DSA levels were more likely to experience a 30% loss of baseline eGFR (Figure 3E, *p* = 0.046). Thus, patients who developed new or increased DSA levels following COVID-19 diagnosis experienced greater late loss of renal function.

### Kidney Transplant Patients Who Developed New or Increased Levels of Donor-Specific Antibodies Received Distinct Medical Management

Our data showed a substantial proportion of KTRs hospitalized with COVID-19 went on to develop new or increased DSA levels that were associated with late allograft dysfunction. Given the temporal association between infection and DSA responses, we hypothesized that antiviral and immunosuppressive therapies would be associated with DSA events following infection. To test this hypothesis, we assessed inpatient medications, vaccination history, and immunosuppressive regimens for the entire cohort (*n* = 112, Table 4). We found that patients who developed new or increased DSA levels were less likely to receive remdesivir (33% vs. 65%, *p* = 0.02, Table 4), which was notable considering rates of AKI were similar between groups. Patients with new or increased DSA levels were less likely to have received at least one dose of a COVID-19 vaccination prior to infection (0% vs. 28%, *p* = 0.018, Table 4) and were admitted earlier in the pandemic. Among the 22 KTRs that had been vaccinated prior to infection, the median time from the first vaccine dose to infection was 137 days (Supplementary Table S4). 16 (72%) received a second vaccine dose ≥3 weeks prior to infection. Patients with new or increased DSA levels had higher pre-admission immunosuppressant doses and presented with lower absolute lymphocyte counts, consistent with more recent transplantation and more intensive immunosuppression. However, inpatient percentage reduction in immunosuppression was not significantly different across any immunosuppressant between groups. In addition, there was no significant difference in the receipt of convalescent plasma between groups. Thus, patients who did not develop new or increased levels of DSA were more likely to (1) have received remdesivir, (2) have received at least one dose of a COVID-19 vaccine prior to infection, or (3) have lower doses of maintenance immunosuppression at the time of admission.

**TABLE 4:** Comparison of medical management based upon the development of new or increased DSA responses following COVID-19.

	DSA negative/unchanged/unknown, N = 97	DSA new/increased, N = 15	*p*-value
**Antimicrobial therapies**			
**Convalescent plasma**	3 (3.1%)	1 (6.7%)	0.4
**Remdesivir**	63 (65%)	5 (33%)	0.020
**Any antibiotics while inpatient**	68 (70%)	9 (60%)	0.6
**Vaccine-related**			
**Time from EUA to COVID-19 diagnosis (days)**	38 (−37, 234)	−57 (−132, 4)	0.001
**COVID-19 diagnosis occurred after EUA**	64 (66%)	6 (40%)	0.053
**At least one vaccine dose prior to COVID-19 infection**	22 (28%)	0 (0%)	0.018
**Home immunosuppressive regimen at time of admission**			
**Home immunosuppression drug regimen**			0.2
Other	14 (14%)	0 (0%)	
Tac + MMF (Steroid Free)	6 (6.2%)	0 (0%)	
Tac + MMF + Prednisone	77 (79%)	15 (100%)	
**Home prednisone dose (mg)**	10.00 (7.50, 10.00)	10.00 (10.00, 10.00)	0.006
**Home tacrolimus dose (mg)**	4.0 (3.0, 7.0)	10.0 (4.0, 11.5)	0.043
**Home MMF dose (mg)**	1,000 (750, 1,500)	1,500 (1,260, 2,000)	0.009
**Inpatient immunosuppression doses**			
**Average daily inpatient steroid dose (prednisone equivalent mg)**	28 (11, 40)	20 (11, 35)	0.4
**Average daily inpatient tacrolimus dose (mg)**	1.31 (0.38, 2.85)	2.50 (0.22, 4.00)	0.4
**Median inpatient tacrolimus level (ng/ml)**	5.03 (3.15, 7.17)	6.70 (4.70, 7.60)	0.3
**Average daily inpatient MMF dose (mg)**	524 (344, 1,000)	646 (34, 1,034)	0.8
**Inpatient immunosuppression as a percentage of home regimen**			
**Percentage of home prednisone dose (%)**	358 (123, 497)	150 (106, 303)	0.10
**Percentage of home tacrolimus dose (%)**	33 (13, 55)	37 (5, 57)	>0.9
**Percentage of home MMF dose (%)**	52 (38, 70)	44 (2, 65)	0.2
**Immune parameters**			
**Admission C-reactive protein (mg/L)**	88 (36, 149)	87 (48, 111)	>0.9
**Admission absolute lymphocytes (10^3 cells/uL)**	0.56 (0.28, 0.81)	0.27 (0.18, 0.45)	0.083

Cell values presented as median (IQR) for continuous variables and n (%) for categorical variables. Pearson’s Chi-squared test was used to compare percentages between categorical variables when all expected cell counts were ≥5, and Fisher’s exact test was used when any expected cell count was <5. Group mean ranks were compared with Mann-Whitney tests. DSA, donor-specific antibody. EUA, emergency-use authorization for BNT-162b2 (Pfizer/BioNTech) on 11 December 2020. Tac, tacrolimus. MMF, mycophenolate mofetil.

### Vaccination Was Associated With Protection From New or Increased Donor-Specific Antibodies

**FIGURE 4:**
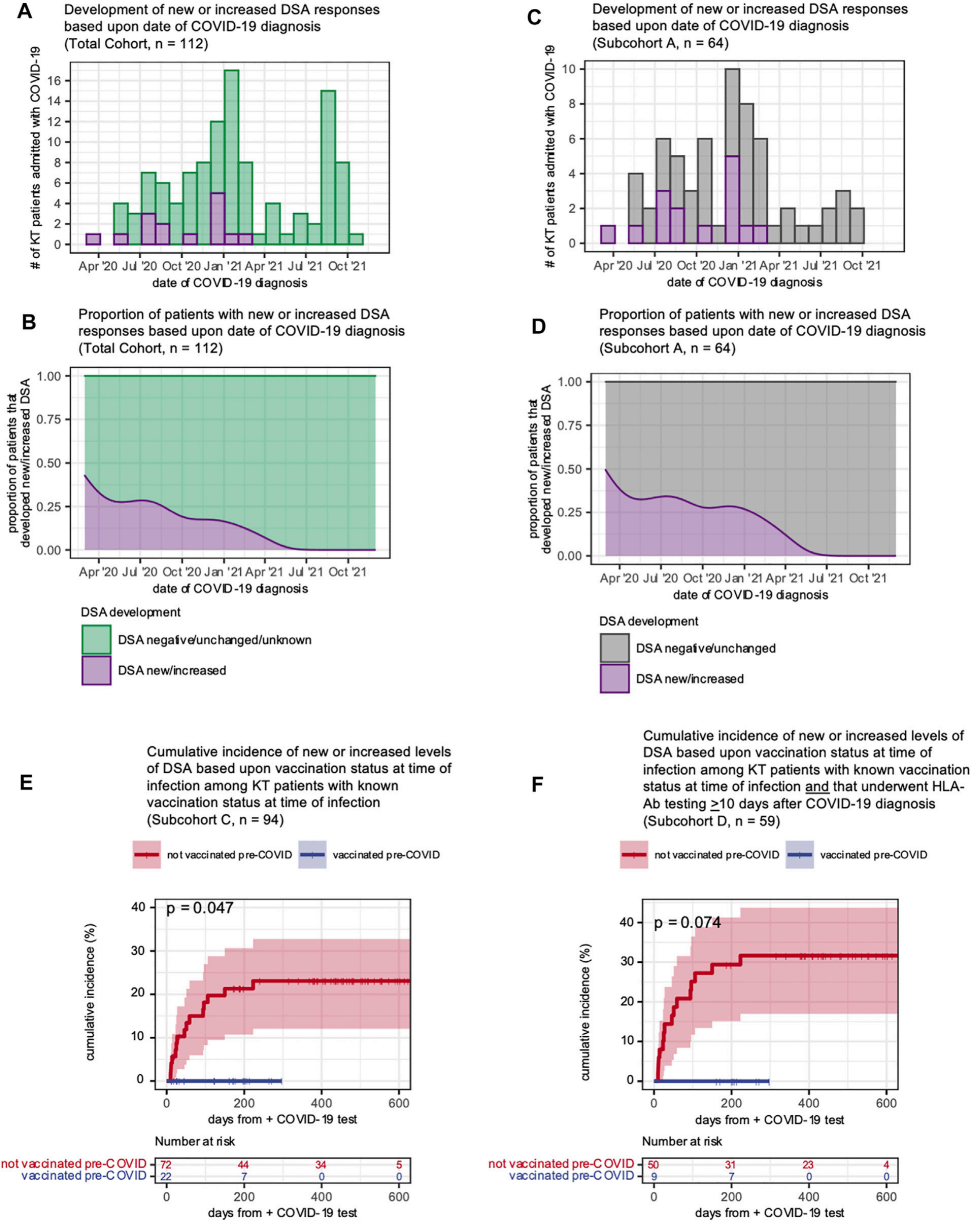
Vaccinated KT patients were less likely to develop new or increased DSA responses following COVID-19 infection. **(A–D)** Development of new or increased DSA responses shown as histograms **(A,C)** displaying the distribution of patients over the study period and smoothed kernel density estimates **(B,D)** that reflect the proportion of patients admitted over time who subsequently developed a new or increased DSA response. Panels **(A,B)** show all KT patients (*n* = 112) hospitalized with a COVID-19 diagnosis over the study period and include patients who developed a new or increased DSA response after COVID-19 (purple) and patients whose DSA status was negative, unchanged or unknown (green). Panels **(C,D)** show subcohort A (patients with HLA-Ab testing ≥10 days after COVID-19 diagnosis) with a new or increased DSA response after COVID-19 (purple) and patients whose DSA status was negative or unchanged (gray). **(E)** The cumulative of incidence of new or increased DSA levels based upon the patient vaccination status at the time of COVID-19 diagnosis. Data shown for *n* = 94 patients (subcohort C) for whom vaccination status was known at the time of admission. **(F)** The cumulative of incidence of new or increased DSA levels based upon the patient vaccination status at the time of COVID-19 diagnosis. Data shown for *n* = 59 patients (subcohort D) for whom vaccination status was known at the time of admission and that had post-COVID-19 HLA-Ab testing. Curves in **(E,F)** were estimated using the Kaplan-Meier method and analyzed with the logrank test.

Given that vaccination status and development of new or increased DSA responses were inversely correlated, we analyzed the temporal relationship between COVID-19 admission date and whether an individual developed a new or increased DSA response. All patients who developed new or increased DSA responses were admitted before 1 March 2021, and none had been vaccinated prior to COVID-19 infection (Figure 4A). The proportion of KTRs who developed new or increased DSA declined as the pandemic progressed (Figure 4B). We found these same relationships for patients with HLA-Ab testing ≥10 days after COVID-19 (subcohort A, Figures 4C,D). Next, using a time-to-event analysis for the entire cohort, we found that patients who had received at least one vaccine dose were significantly less likely to develop a new or increased DSA response (Figure 4E, *p* = 0.047). As a sensitivity analysis, we performed the same comparison for those individuals for whom HLA-Ab testing post-COVID-19 was available (subcohort D). Importantly, we observed a similar inverse correlation between vaccination status and likelihood of developing a new or increased DSA response (Figure 4F, *p* = 0.074). Of note, although patients that were vaccinated and subsequently admitted had shorter follow-up time given their more recent admissions, these patients had still had a median follow-up time of 151 days, and our data (Figure 2B) suggests that the majority of post-COVID-19 DSA events occur within this follow-up interval. Thus, KTRs vaccinated prior to infection or admitted later in the course of the pandemic were less likely to develop new or increased DSA post-COVID-19.

### Secular Trends in COVID-19 Severity and Management

**FIGURE 5:**
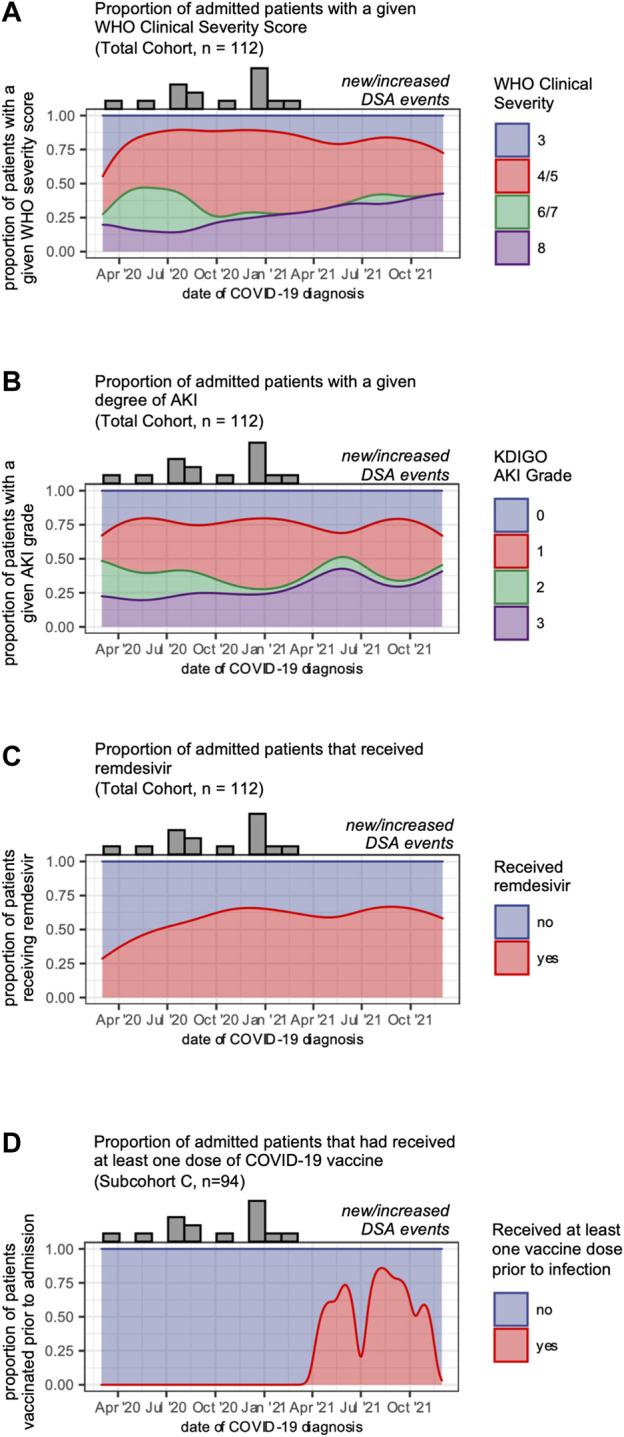
Secular trends in COVID-19 disease severity and management. **(A)** Disease severity of patients admitted over the study period shown as smoothed kernel density estimates that reflect the proportion of patients admitted over time who reached a given WHO clinical severity score. **(B)** Degree of acute kidney injury for patients admitted over the study period shown as smoothed kernel density estimates that reflect the proportion of patients admitted over time who reached a given grade of AKI. **(C)** Remdesivir use for patients admitted over the study period shown as smoothed kernel density estimates that reflect the proportion of patients admitted over time who received remdesivir. **(D)** Vaccination status for patients admitted over the study period shown as smoothed kernel density estimates that reflect the proportion of patients admitted over time who had received at least one dose of a COVID-19 vaccine prior to admission. Along the top of each graph are counts of the number of admitted patients who developed a new or increased DSA response in each month.

Our data showed that in addition to transplant-specific risk factors, vaccination and remdesivir use were associated with protection from new or increased DSA responses. As disease management, variant prevalence, and outcomes changed during the pandemic, we asked whether other secular trends might be associated with the declining risk of DSA events in this cohort. We observed a trend towards slightly greater disease severity as the pandemic progressed, as measured by WHO clinical severity and the proportion of patients experiencing a grade 3 AKI (Figures 5A,B). We also found that remdesivir use increased during the fall of 2020 and stayed stable following that time (Figure 5C), and that vaccination increased in 2021 (Figure 5D). These findings support the notion that the declining incidence of DSA events is not explained by declining disease severity, and instead may be associated with trends in management.

## Discussion

We showed that new or increased DSA responses in KTRs following admission with COVID-19 was relatively common, with a cumulative incidence of 17% at 8 months following infection. This DSA response was associated with impaired allograft function. We further found that the risk of developing a new or increased DSA response was significantly higher in recently transplanted patients. By contrast, COVID-19 vaccination prior to infection as well as the administration of remdesivir during infection were associated with protection from the development of a new or increased DSA response.

Our study is not the first to examine DSA changes in KTRs following COVID-19 infection (5). Masset *et al.* showed that 8% of KTRs hospitalized with COVID-19 developed new or increased levels of DSA within a median follow-up of 45 days (5). Consistent with the Masset et al. study (5), we observed that recent transplant, younger age, and pre-COVID-19 DSA were each associated with an increased risk for a change in DSA levels. By extending the follow-up time, we found the cumulative incidence of a new or increased DSA response was 17% within 8 months of COVID-19 diagnosis. Importantly, these changes in DSA responses occurred throughout those 8 months. Interestingly, of the 15 individuals who experienced a change in their DSA response following infection, 10 (66%) developed a new DSA specificity, considerably higher than the expected incidence of *de novo* DSA in a general KT population (19). Our data also show that these DSA responses do not only occur immediately following the acute infection. We speculate that these delayed alloimmune events may be related to ongoing inflammation or tissue injury. Given that SARS-CoV-2 may persist in the immunocompetent human host for at least 4 months (20) and that immunosuppressed patients may have relatively prolonged viral shedding (21), extended infection may promote alloimmunity, either through chronic immune activation (22) or direct allograft injury (23–25).

Consistent with data showing SOT patient outcomes improved as clinical practices were refined during the pandemic (26), our temporal analysis revealed that patients admitted in the post-EUA era were significantly less likely to develop new or increased DSA responses. This reduction in alloimmunity was associated with COVID-19 vaccination pre-infection as well as with administration of remdesivir during acute infection. Given the lack of association between DSA responses and acute disease severity or acute kidney injury, these data suggest that interventions like vaccination or remdesivir treatment that decrease viral replication (27, 28) may be associated with a reduced risk of alloimmunity.

Our data suggest three potential clinical considerations for KTRs in the ongoing COVID-19 pandemic. First, for KTRs hospitalized with COVID-19, new or increased DSA formation was relatively common, particularly among non-vaccinated patients. Given the association between DSA changes and impaired late allograft function, it may be prudent to consider more frequent DSA surveillance in this population, particularly among patients with increased alloimmune risk (a recent transplant or pre-COVID-19 DSA). Second, given the vulnerability of recently transplanted patients, the impaired efficacy of vaccination in the immunosuppressed patient (29), and concerns surrounding vaccination policies (30, 31), our findings suggest that pre-transplant vaccination in this cohort has the potential to be beneficial in reducing the incidence of alloimmune responses post-COVID-19. Importantly, our data provide no evidence that vaccines promote alloimmunity. Third, we observed that administration of remdesivir was associated with a reduced risk of developing a new or increased DSA response. It will be important to assess the efficacy of new antiviral therapies in this vulnerable KT patient population—both for reducing disease severity as well as for any association with abrogating alloimmune responses.

Our study was conducted at a single center and was limited to KTRs hospitalized with COVID-19. Although we did not genotype SARS-CoV-2 strains, we think it is unlikely that changing variants could explain our findings as all admissions predated Omicron’s emergence (32), and pre-Omicron variants have shown similar disease severity (33) and similar tropism for the kidney (34). Our analysis of other secular trends indicate that while management features changed, disease severity largely did not. Although we were able to document vaccine administration, we did not have pre-disease antiviral serologic data as a surrogate for vaccine efficacy. Since most testing and sample collection in our study group was guided by clinical care rather than research protocols, HLA-Ab testing was likely biased towards patients with increased immune risk or evidence of allograft dysfunction. As we did not have equivalent samples across the entire cohort, we may have underestimated the incidence of DSA events in untested patients. Finally, although we found an association between DSA events and loss of eGFR, we lacked sufficient allograft biopsy data to correlate DSA events with histopathological lesions. Importantly, the conclusions that were reached for the whole cohort were well-supported by the subcohort-based sensitivity analyses. Finally, the relatively small number of events for our outcomes of interest limited our ability to perform multivariate analysis, and thus our findings should be interpreted as correlational.

Despite the limitations associated with our single center study, we demonstrated that KTRs hospitalized with COVID-19 often developed new or increased DSA responses. These changes in DSA status were associated with impaired late allograft function. Vaccination and administration of remdesivir were each associated with protection from the development of a new or enhanced DSA response. Recent transplant and the presence of pre-COVID-19 DSA were positively associated with new or increased DSA responses. These data, which identify patients at higher risk of developing humoral alloimmune responses, suggest a role for more intensive HLA-Ab surveillance and provide indirect evidence of the possible benefits of preventing alloimmunity in SARS-CoV-2-infected KTRs through vaccination or antiviral therapies.

## Data Availability

The raw data supporting the conclusions of this article will be made available by the authors, without undue reservation.

## Abbreviations

KT, kidney transplant; KTR, kidney transplant recipient; nonSOT, non-solid organ transplant; DSA, donor-specific antibody; UAB, University of Alabama at Birmingham; HLA-Ab, anti-HLA antibody; MFI, mean fluorescence intensity; %SA, percentage single antigen beads; eGFR, estimated glomerular filtration rate; CKD-EPI, Chronic Kidney Disease Epidemiology Collaboration; AKI, Acute kidney injury; KDIGO, Kidney Disease Improving Global Outcomes; sCr, serum creatinine; WHO, World Health Organization; IQR, interquartile range; KM, Kaplan-Meier; AMR, antibody-mediated rejection; EUA, emergency use authorization.
