# Supplementary figures and images for: COVID-19 Vaccination and Remdesivir are Associated With Protection From New or Increased Levels of Donor-Specific Antibodies Among Kidney Transplant Recipients Hospitalized With COVID-19
Source: Transpl Int. 2022 Jul 19;35:10626. doi: 10.3389/ti.2022.10626 (PMC9343962; doi:10.3389/ti.2022.10626)

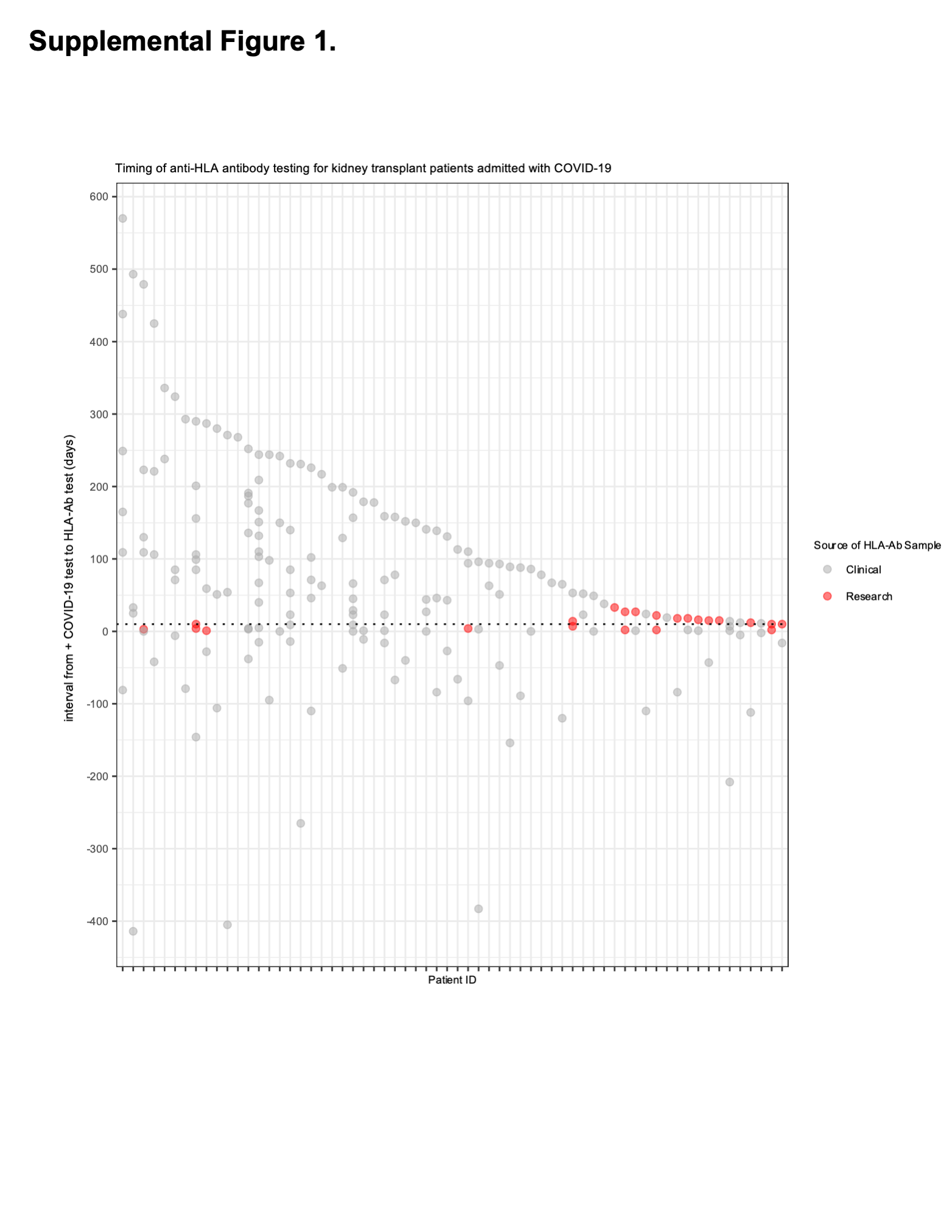

Supplement: Supplementary file 1 [file Image1.TIFF]
