# Supplementary material for: COVID-19 Vaccination and Remdesivir are Associated With Protection From New or Increased Levels of Donor-Specific Antibodies Among Kidney Transplant Recipients Hospitalized With COVID-19
Source: Transpl Int. 2022 Jul 19;35:10626. doi: 10.3389/ti.2022.10626 (PMC9343962; doi:10.3389/ti.2022.10626)
Supplement: Supplementary file 2 [file DataSheet1.docx]

**Supplemental Figure 1. Timing of HLA-Ab testing for KT patients.**

Each vertical lane along the x-axis indicates a unique patient from subcohort A (n=64 patients). Points are plotted at the time of HLA-Ab testing relative to the day of COVID-19 diagnosis. Gray points indicate HLA-Ab testing that was ordered by a clinician. Red points indicate serum specimens that were collected for research purposes and were subsequently tested for HLA-Abs. n=193 total specimens. The dashed line indicates 10 days after COVID-19 diagnosis.

**Supplemental Table 1. Magnitude of MFI increases for five patients whose pre-existing DSA increased following COVID-19.**

| Patient ID | Immuno-  dominant HLA | timing of first DSA measurement relative to + COVID-19 test (days) | timing of second DSA measurement relative to + COVID-19 test (days) | MFI  (first measurement) | MFI  (second measurement) | Absolute Increase in MFI | % Increase in MFI |
| --- | --- | --- | --- | --- | --- | --- | --- |
| Patient 042 | DRB4*01:03 | 3 | 223 | 20147 | 25227 | 5080 | 25.2% |
| Patient 044 | B*35:01 | 3 | 96 | 4802 | 7259 | 2457 | 51.2% |
| Patient 046 | DQA1*05:03 + DQB1*03:01 | -112 | 12 | 1826 | 7992 | 6166 | 337.7% |
| Patient 068 | DQA1*03:02 + DQB1*03:02 | -42 | 106 | 2421 | 4856 | 2435 | 100.6% |
| Patient 085 | DQA1*05:01 + DQB1*02:01 | -106 | 51 | 2109 | 3243 | 1134 | 53.8% |

**Supplemental Table 2.** **Comparison of disease severity and renal function based upon the development of a new or increased DSA response following COVID-19 for subcohort B.**

Cell values presented as median (IQR) for continuous variables and n (%) for categorical variables. Pearson’s Chi-squared test was used to compare percentages between categorical variables when all expected cell counts were >5, and Fisher’s exact test was used when any expected cell count was <5. Group mean ranks were compared with Mann-Whitney tests. DSA, donor-specific antibody. WHO, World Health Organization. ECMO, extracorporeal membrane oxygenation. AKI, acute kidney injury. KDIGO, Kidney Disease Improving Global Outcomes. CRRT, continuous renal replacement therapy. HD, hemodialysis. eGFR, estimated glomerular filtration rate

|  | **DSA negative/unchanged**, **unknown,**  N = 46 | **DSA new/increased**,  N = 10 | **p-value** |
| --- | --- | --- | --- |
| **Measures of disease severity** | | | |
| **Length of stay (days)** | 6 (4, 11) | 6 (3, 10) | 0.7 |
| **ICU requirement** | 8 (17%) | 2 (20%) | >0.9 |
| **Highest WHO COVID-19 disease severity scale** |  |  | 0.2 |
| 3 (no supplemental oxygen) | 8 (17%) | 3 (30%) |  |
| 4 (supplemental oxygen via nasal cannula) | 23 (50%) | 4 (40%) |  |
| 5 (supplemental oxygen via high-flow nasal cannula, BiPap, or CPAP) | 11 (24%) | 1 (10%) |  |
| 6 (endotracheal intubation and mechanical ventilation) | 1 (2.2%) | 2 (20%) |  |
| 7 (endotracheal intubation and mechanical ventilation + vasopressor support or ECMO) | 3 (6.5%) | 0 (0%) |  |
| **Discharge disposition** |  |  | >0.9 |
| Home | 39 (85%) | 9 (90%) |  |
| Long-term care facility | 7 (15%) | 1 (10%) |  |
| **Renal function** | | | |
| **AKI Grade (KDIGO)** |  |  | 0.10 |
| 0 | 16 (35%) | 3 (30%) |  |
| 1 | 24 (52%) | 4 (40%) |  |
| 2 | 2 (4.3%) | 3 (30%) |  |
| 3 | 4 (8.7%) | 0 (0%) |  |
| **CRRT/HD while inpatient** | 2 (4.3%) | 0 (0%) | >0.9 |
| **Baseline eGFR (mL/min/1.73 m2)** | 49 (33, 61) | 58 (39, 65) | 0.5 |
| **Absolute change in eGFR on 90+ day follow-up** | 1 (-8, 7) | -14 (-17, -5) | 0.013 |
| **Percentage change in eGFR on 90+ day follow-up** | 4 (-19, 17) | -29 (-56, -9) | 0.005 |
| **At least 30% loss in eGFR on 90+ day follow-up** |  |  | 0.027 |
| <30% | 42 (91%) | 6 (60%) |  |
| >=30% | 4 (8.7%) | 4 (40%) |  |

**Supplemental Table 3.** **Comparison of disease severity and renal function based upon the development of a new or increased DSA response following COVID-19 for patients in subcohort B that also had post-COVID-19 HLA-Ab testing.**

Cell values presented as median (IQR) for continuous variables and n (%) for categorical variables. Pearson’s Chi-squared test was used to compare percentages between categorical variables when all expected cell counts were >5, and Fisher’s exact test was used when any expected cell count was <5. Group mean ranks were compared with Mann-Whitney tests. DSA, donor-specific antibody. WHO, World Health Organization*^.^* ECMO, extracorporeal membrane oxygenation. AKI, acute kidney injury. KDIGO, Kidney Disease Improving Global Outcomes. CRRT, continuous renal replacement therapy. HD, hemodialysis. eGFR, estimated glomerular filtration rate

|  | **DSA negative/unchanged**, N = 32 | **DSA new/increased**,  N = 10 | **p-value** |
| --- | --- | --- | --- |
| **Measures of disease severity** | | | |
| **Length of stay (days)** | 7 (4, 14) | 6 (3, 10) | 0.5 |
| **ICU requirement** | 7 (22%) | 2 (20%) | >0.9 |
| **Highest WHO COVID-19 disease severity scale** |  |  | 0.2 |
| 3 | 4 (12%) | 3 (30%) |  |
| 4 | 17 (53%) | 4 (40%) |  |
| 5 | 7 (22%) | 1 (10%) |  |
| 6 | 1 (3.1%) | 2 (20%) |  |
| 7 | 3 (9.4%) | 0 (0%) |  |
| **Discharge disposition** |  |  | >0.9 |
| Home | 27 (84%) | 9 (90%) |  |
| Long-term care facility | 5 (16%) | 1 (10%) |  |
| **Renal function** | | | |
| **AKI Grade (KDIGO)** |  |  | 0.2 |
| 0 | 10 (31%) | 3 (30%) |  |
| 1 | 16 (50%) | 4 (40%) |  |
| 2 | 2 (6.2%) | 3 (30%) |  |
| 3 | 4 (12%) | 0 (0%) |  |
| **CRRT/HD while inpatient** | 2 (6.2%) | 0 (0%) | >0.9 |
| **Baseline eGFR (mL/min/1.73 m2)** | 47 (35, 61) | 58 (39, 65) | 0.5 |
| **Absolute change in eGFR on 90+ day follow-up** | -2 (-11, 6) | -14 (-17, -5) | 0.039 |
| **Percentage change in eGFR on 90+ day follow-up** | -5 (-22, 11) | -29 (-56, -9) | 0.015 |
| **At least 30% loss in eGFR on 90+ day follow-up** |  |  | 0.075 |
| <30% | 28 (88%) | 6 (60%) |  |
| >=30% | 4 (12%) | 4 (40%) |  |

**Supplemental Table 4.** **Details of vaccination prior to COVID-19 infection.**

Cell values presented as median (IQR) for continuous variables and n (%) for categorical variables

|  | **N = 22** |
| --- | --- |
| **Received at least one dose before COVID-19 infection** | 22 (100%) |
| **Was 21+ days beyond 2nd dose at time of COVID-19 infection** | 16 (73%) |
| **Number of doses received prior to COVID-19 infection** |  |
| 1 | 4 (18%) |
| 2 | 18 (82%) |
| **Number of days between those receiving first dose only and COVID-19 infection (n = 22)** | 137 (75, 185) |
| **Number of days between those receiving second dose and COVID-19 infection (n = 18)** | 138 (78, 163) |
| Specific date of pre-infection vaccination unknown | 4 |
| **Manufacturer for all doses** |  |
| BNT-162b2 (Pfizer/BioNTech) | 10 (45%) |
| Janssen Ad26 (J&J) | 2 (9.1%) |
| mRNA-1273 (Moderna) | 9 (41%) |
| Unknown | 1 (4.5%) |
